# Genetic diversity of transmission-blocking vaccine candidate antigens Pvs25 and Pvs28 in *Plasmodium vivax* isolates from China

**DOI:** 10.1186/s12879-022-07931-0

**Published:** 2022-12-16

**Authors:** Siqi Wang, Peng Tian, Shigang Li, Hui Liu, Xiangrui Guo, Fang Huang

**Affiliations:** 1 National Institute of Parasitic Diseases, Chinese Center for Disease Control and Prevention, Shanghai, 200025 China; 2 Chinese Center for Tropical Diseases Research, Shanghai, 200025 China; 3 NHC Key Laboratory of Parasite and Vector Biology, National Institute of Parasitic Diseases, Chinese Center for Disease Control and Prevention, Shanghai, 200025 China; 4 WHO Collaborating Centre for Tropical Diseases, National Center for International Research on Tropical Diseases, Shanghai, 200025 China; 5 Yunnan Institute of Parasitic Diseases, Pu’er, 665000 China; 6 Yingjiang County Center for Disease Control and Prevention, Yingjiang, 679300 China; 7 Shanghai Municipal Center for Disease Control and Prevention, Shanghai, 200336 China

**Keywords:** *Plasmodium vivax*, *pvs25*, *pvs28*, Genetic diversity, China

## Abstract

**Background:**

Transmission-blocking vaccines (TBVs) target the sexual stages of malaria parasites to reduce or interrupt the transmission cycle in human and mosquito populations. The genetic diversity of TBVs candidate antigens, Pvs25 and Pvs28, in *Plasmodium vivax* could provide evidence for the development of TBVs.

**Methods:**

Dry blood spots from *P. vivax* patients were collected from Dandong, Suining, Hainan, Nyingchi, Tengchong, and Yingjiang in China. The *pvs25* and *pvs28* genes were amplified and sequenced. The genetic diversity of *pvs25* and *pvs28* were analyzed using DNASTAR, MEGA6, and DnaSP 5.0 programs.

**Results:**

A total of 377 samples were collected, among which 324 and 272 samples were successfully amplified in the *pvs25* and *pvs28* genes, respectively. Eight haplotypes were identified in Pvs25, for which the predominant mutation was I130T with 100% prevalence. A variety of 22 haplotypes in Pvs28 were identified. The number of GSGGE/D repeats of Pvs28 was a range of 4–8, among which, high (7–8) and low (4–5) copy numbers of tandem repeats were found in haplotypes H2 and H17, respectively. The nucleotide diversity of *pvs28* (π = 0.00305 ± 0.00061) was slightly higher than that of *pvs25* (π = 0.00146 ± 0.00007), thus they were not significantly different (*P* > 0.05). The Tajima's D value of *pvs25* was positive whereas *pvs28* was negative, which indicated that both genes were affected by natural selection.

**Conclusion:**

The genetic diversity of *pvs25* and *pvs28* genes in China was relatively limited, which provided valuable information for TBVs design and optimization.

## Introduction

There were an estimated 241 million malaria cases globally in 2020, an increase from 2019 and approximately 4.5 million cases were caused by *Plasmodium vivax*, which was a decline from 2000, but remains a serious public health concern [1]. Among the five human malaria species, *P. vivax* is not as harmful as *Plasmodium falciparum*, which causes malignant malaria globally, although it is the most widespread outside of Africa. *P. vivax* represents the most prevalent relapse form of malaria constitutes a major obstacle for elimination efforts. China was certified malaria free in June of 2021; however, the risk of malaria re-establishment caused by imported malaria still exists. Interrupting the spread of imported drug-resistant *P. vivax* remains one of the major challenges during the post-elimination phase.

Transmission-blocking vaccines (TBVs) target the sexual stages of malaria parasites to reduce or interrupt the transmission cycle in human and mosquito populations [2, 3]. TBVs are primarily mediated by antibodies to *Plasmodium* surface proteins and act on the mosquito midgut [4]. To date, effective target antigens of TBVs have been identified and demonstrate good immune-blocking activity [5]. Among these antigens, the surface proteins P25 and P28 are expressed on zygotes and mature ookinetes [6]. The most striking feature of these proteins is that they have four epidermal growth factor (EGF)-like and cysteine-rich domains [7]. Previous studies had shown that the P25 protein was expressed earlier than the P28 protein associated with parasite development in mosquitoes [8]. Among the TBVs candidate proteins, the *P. vivax* proteins Pvs25 and Pvs28 were most concerned. Antisera against both recombinant Pvs25 and Pvs28 can recognize the corresponding antigens expressed by the zygotes/ookinetes, thereby inhibiting the development of mosquito oocysts [9]. When these antisera were diluted with the blood of *P. vivax* infected chimpanzees, a significant reduction in the number of oocysts was observed; however, anti-Pvs25 antiserum achieved a significantly greater blockade compared to the anti-Pvs28 antiserum [9]. Pvs25 and Pvs28 antisera were also identified in the *P. vivax* isolates from Thailand, which could recognize the corresponding molecules expressed by the parasite and block its spread [10]. Pvs25 and Pvs28 were more polymorphic than the *P. falciparum* homologous products, but the antigenic polymorphism of Pvs25 was more limited than that of Pvs28 [11]. Pvs25 has been considered as the leading *vivax* malaria TBVs candidate. Additionally, although the Pvs28 was expressed later in mosquitoes, they were all conservative substitutions [11].

Until now, the genetic diversity of the *pvs25* and *pvs28* genes has been reported in malaria-endemic countries, including Mexico [12], Iran [13], Bangladesh [14], South Korea [15, 16], India [17], Myanmar [18], Thailand [10], and Yunnan Province in China [19]. In the present study, the genetic diversity of the TBVs candidate antigens, Pvs25 and Pvs28, was detected and analyzed in *P. vivax* isolates collected from six different locations in China.

## Materials and methods

### Study site

**Fig. 1:**
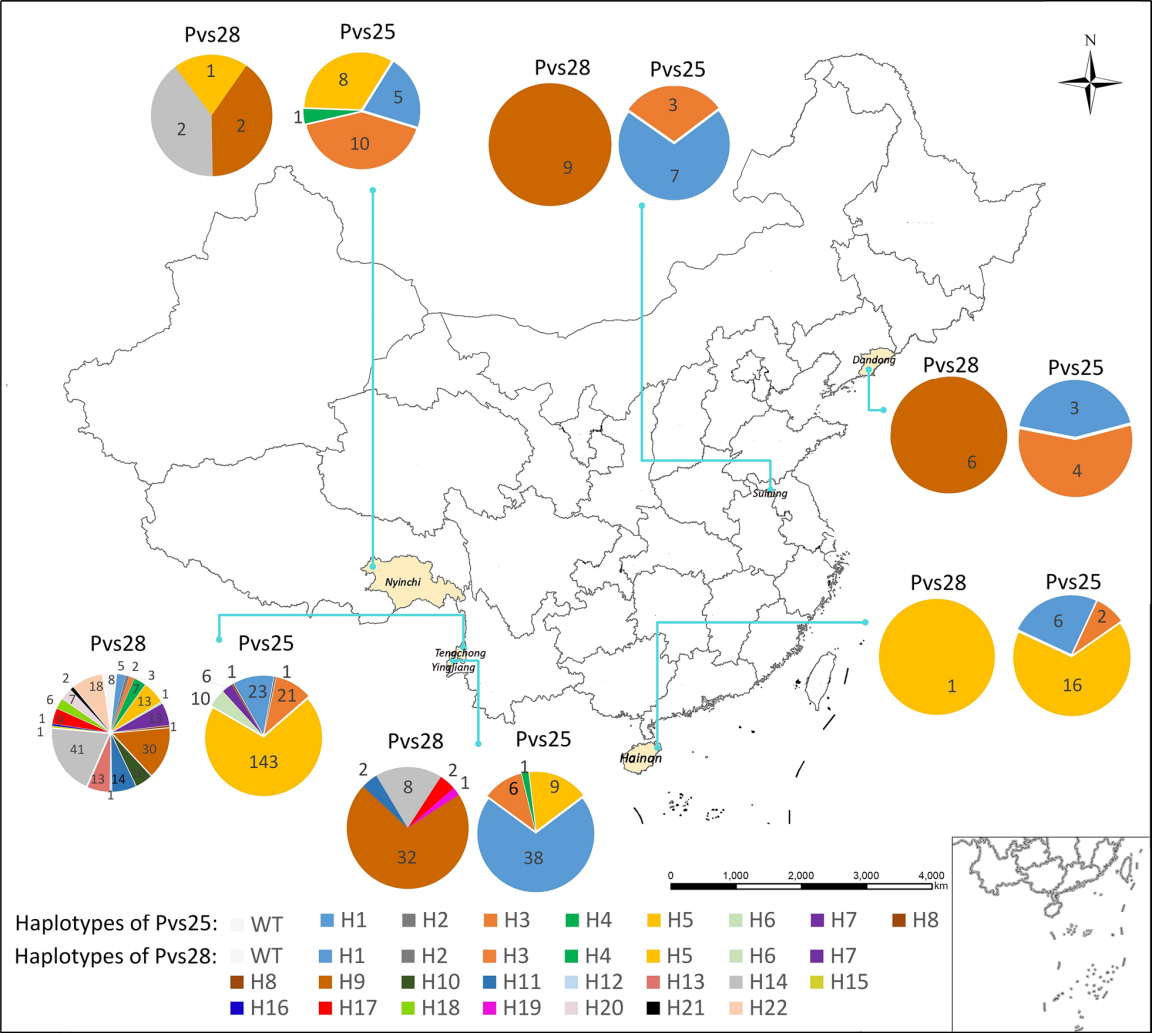
Haplotype distribution of Pvs25 and Pvs28 in different regions of China, 2009–2020. *Only one isolate from Hainan was the H5 haplotype of Pvs28, six isolates in Dandong, and nine isolates in Suining were H9 haplotypes of Pvs28

Blood samples were collected from Dandong, Suining, Nyingchi, Tengchong, Yingjiang, and Hainan Island. Dandong city in Liaoning Province is in northeastern China. Suining county in Jiangsu Province is located in central China. Nyingchi city in Tibet is located in western China. Tengchong and Yingjiang counties in Yunnan Province are located in southwestern China along the China-Myanmar border. Hainan Island is the southernmost part of China (Fig. 1).

### Sample collection

*P. vivax* isolates were collected between 2009 and 2020 from six different geographic sites in China. Finger-prick blood was spotted on filter paper (Whatman™ 903, GE Healthcare, USA) and dried. All patients were diagnosed as *P. vivax* using a microscopic examination and reconfirmed using polymerase chain reaction (PCR) by amplifying the small-subunit rRNA gene of *Plasmodium* spp [20]. Written informed consent was obtained from the patients prior to blood collection. This study was approved by the Ethical Review Committee of National Institute of Parasitic Diseases, Chinese Center for Disease Control and Prevention.

### Genomic DNA extraction and amplification

Genomic DNA was extracted using QIAamp DNA Blood Mini Kits (Qiagen, USA) following the manufacturer’s instructions. The Salvador I (Sal-I) strain of *P. vivax* was used as the reference sequences for *pvs25* and *pvs28* genes. Nucleotide sequence of *pvs25* and *pvs28* were available in the GenBank databases under the accession numbers AF083502 and AF083503, respectively. The full-length target genes *pvs25* and *pvs28* were amplified by nested PCR using primer pairs: pvs25F (5′-CACTTAGCCAAAATGAACTC-3′) and pvs25R (5′-AAAGGACAAGCAGGATGATA-3′) for *pvs25*; pvs28F (5′-CTACCACAGCTTGCTGTTCC-3′) and pvs28R (5′-TGACATCATGAAGAAGGCG-3′) for *pvs28*. [11, 17]. Bidirectional sequencing of PCR products was performed by Oebiotech Technology Co., Ltd.

### Data analysis

The nucleotide and deduced amino acid sequences were analyzed using Bioedit and SeqMan in the DNASTAR package (DNASTAR, Madison, WI, USA). The difference of the amino acid substitutions and haplotype of Pvs25 and Pvs28 among different sites in China were statistically analyzed. All sequences were compared with the Sal-1 strain and the amino acid mutations were shown in bold in the results and tables. The haplotypes were determined based on the type of amino acid substitutions of the obtained sequences, and the number of GSGGE/D repeats was calculated by comparing the haplotypes with the reference sequences of Sal-I strain. The values of segregating sites (S), average number pair-wise nucleotide differences (K), haplotype diversity (Hd), and nucleotide diversity (π) were calculated using DnaSP (version 5.0) [21]. The Tajima’s D test [22] was analyzed to evaluate the neutral theory of natural selection using DnaSP. The number of synonymous (dS) and non-synonymous (dN) substitutions were estimated and compared using a Z-test with the program MEGA6 (*P* < 0.05). The Nei and Gojobori’ s method [23, 24] was used to test the null hypothesis of strict neutrality with the Jukes and Cantor correction.

## Results

### Polymorphism of Pvs25

**Fig. 2:**
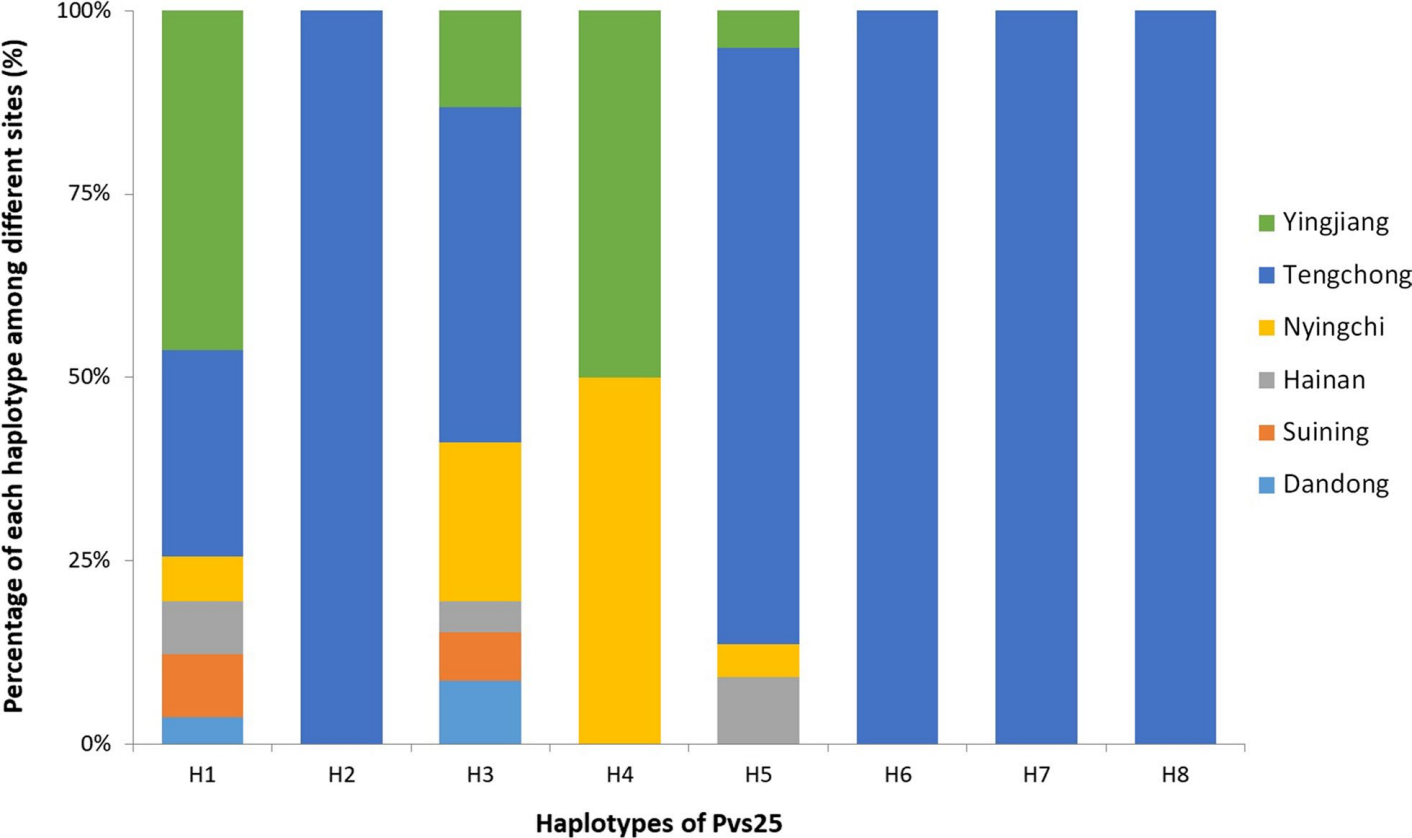
Pvs25 haplotype frequencies of *P. vivax* in different sites in China, 2009–2020

*Pvs25* gene was successfully amplified in 324 *P. vivax* isolates, including seven from Dandong, 10 from Suining, 24 from Hainan, 24 from Nyingchi, 205 and 54 from Tengchong and Yingjiang, respectively. Compared to the reference Sal-I sequence, a variety of point mutations were found in *pvs25* gene, which were all nonsynonymous. A total of 6 amino acid substitutions (D27N, Q87L, E97Q, T100S, I130T, and Q131K) in the Pvs25 protein were identified, which could be classified into 8 different haplotypes (Table 1; Fig. 1). A new amino acid substitution, D27N, was first detected in the first EGF-like domain (EGF-1). The amino acid substitutions of Q87L, E97Q, and T100S were found in the second EGF-like domain (EGF-2). The other two, I130T and Q131K, were detected in the third EGF-like domain (EGF-3). The amino acid substitution of I130T was a predominant variation with 100% prevalence in China (Table 1). Meanwhile, when compared the sequences of Chinese isolates using pv01 strain from Indonesia, both had 130 T mutation, which is common in Asia [19, 25]. Considering the geographical distribution, H5 (DQET**TK**) was the most common haplotype of Pvs25, accounting for 54.3% (Table 1). Two isolates from Tengchong showed the H2 (**N**QET**T**Q) and H8 (**N**Q**Q**T**TK**) haplotypes, respectively (Fig. 2). The H1 (DQET**T**Q) and H3 (DQ**Q**T**T**Q) were the most widely distributed haplotypes, both of which were found in all study sites. The haplotype H4 (DQE**ST**Q) of Pvs25 was found in Yingjiang and Nyingchi each.

**Table 1:** Amino acid variation of the Pvs25 in *P. vivax* isolates from China, 2009–2020

Haplotypes	Number (%)	EGF-1	EGF-2			EGF-3	
		27	87	97	100	130	131
Sal-I	–	D	Q	E	T	I	Q
pv01^*^				**Q**		**T**	
H1	82 (25.3)					**T**	
H2	1 (0.3)	**N**				**T**	
H3	46 (14.2)			**Q**		**T**	
H4	2 (0.6)				**S**	**T**	
H5	176 (54.3)					**T**	**K**
H6	10 (3.1)			**Q**		**T**	**K**
H7	6 (1.9)		**L**	**Q**		**T**	**K**
H8	1 (0.3)	**N**		**Q**		**T**	**K**
Total	324 (100)	–	–	–	–	–	–

EGF, epidermal growth factor-like domain. Dot (•) indicates identical amino acid residues compared to the Sal-I strain. *The pv01 strain from Indonesia was used as another compared sequence, which had the same variation 130T as the isolates from China. Bold letters indicate amino acid variation compared to the Sal-I strain

### Polymorphism of Pvs28

**Fig. 3:**
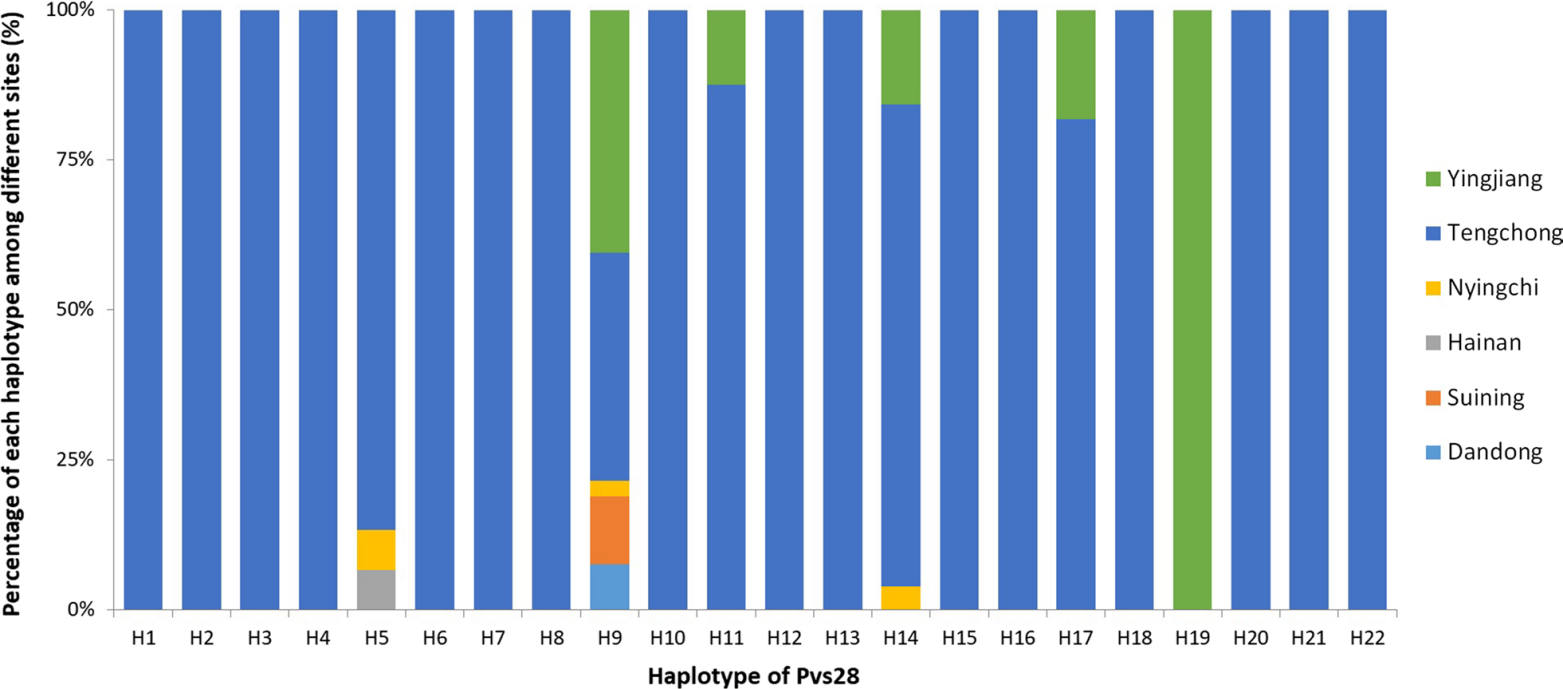
Pvs28 haplotype frequencies of *P. vivax* in different sites in China, 2009–2020

A total of 272 *P. vivax* samples were successfully sequenced in *pvs28* gene, including 6 from Dandong, 9 from Suining, 1 from Hainan, 5 from Nyingchi, 206 from Tengchong, and 45 from Yingjiang. Compared to the Sal-I sequence, a variety of 11 amino acid substitutions were identified, including M52L and A53V in EGF-1, T65K, A81T/V, G95N, L98I/S, and E105K in EGF-2, L116V, D125N, S131N, and T140S in EGF-3. Wild-type were only found in 8 isolates. Twenty-two haplotypes of Pvs28 were identified, which exhibited a greater polymorphic pattern than Pvs25 (Table 2; Fig. 1). The haplotype H9 was predominant, which accounted for 29%, with two amino acid substitutions at M52L and T140S. Moreover, H9 was the most widely distributed haplotype in five sites, except Hainan (Fig. 3). The haplotypes H5, H9, and H14 were associated with Nyingchi isolates (Fig. 1). Additionally, five haplotypes (H9, H11, H14, H17 and H19) were detected in Yingjiang, among which H19 was only found in this location. Two different types of amino acid substitutions were observed at A81T/V and L98I/S in Tengchong.

**Table 2:** Amino acid variation of the Pvs28 in *P. vivax* isolates from China, 2009–2020

Haplotypes	Number (%)	EGF-1		EGF-2					EGF-3				GSGGE/D repeats
		52	53	65	81	95	98	105	116	125	131	140	
Sal-I	–	M	A	T	A	G	L	E	L	D	S	T	6
WT	8 (2.9)												6–7
H1	5 (1.8)	**L**											5–7
H2	2 (0.7)		**V**										6–8
H3	3 (1.1)					**N**							6
H4	7 (2.6)							**K**					5–6
H5	15 (5.5)								**V**				6
H6	1 (0.3)										**N**		6
H7	13 (4.8)											**S**	5–7
H8	1 (0.3)	**L**							**V**				6
H9	79 (29.0)	**L**										**S**	5–7
H10	10 (3.7)		**V**						**V**				6
H11	16 (5.9)		**V**									**S**	6–7
H12	1 (0.3)					**N**		**K**					6
H13	13 (4.8)						**I**		**V**				6
H14	51 (18.8)							**K**	**V**				5–7
H15	1 (0.3)	**L**					**I**		**V**				6
H16	1 (0.3)	**L**	**V**									**S**	6
H17	11 (4.0)	**L**		**K**								**S**	4–6
H18	6 (2.2)	**L**					**S**					**S**	6
H19	1 (0.3)	**L**								**N**		**S**	6
H20	7 (2.6)		**V**		**T**				**V**				6–7
H21	2 (0.7)						**I**		**V**			**S**	6
H22	18 (6.6)	**L**		**K**	**V**							**S**	5–6
Total	272 (100)	–	–	–	–	–	–	–	–	–	–	–	–

EGF, epidermal growth factor-like domain. Dot (•) indicates identical amino acid residues compared to the Sal-I strain. Bold letters indicate amino acid variation compared to the Sal-I strain

There was a range of 4–8 GSGGE/D repeats in Pvs28 (Table 2). One isolate with 7 copies of the GSGGE/D tandem repeats was found in the wild-type Pvs28. Samples from Dandong and Suining contained seven copies of the GSGGE/D tandem repeat, and were classified as haplotype H9. Both high (7–8) and low (4–5) copy numbers of tandem repeats were found in haplotypes H2 and H17, respectively, and both were found in Tengchong (Table 2; Fig. 3). A total of 215 (79.0%) isolates had six copies of the GSGGE/D tandem repeat at the end of the EGF-4 domain, which were consistent with the Sal-I strain.

### Gene polymorphisms of *pvs25* and *pvs28*

The genetic diversity of *pvs25* and *pvs28* genes were analyzed on both successfully sequenced samples (Tables 3, 4). The haplotype diversity (Hd) of Pvs25 and Pvs28 of all samples was 0.621 ± 0.021 and 0.485 ± 0.087, respectively. The average number of *pvs25* nucleotide differences (K = 0.859) was lower than that of *pvs28* (K = 1.679). The nucleotide diversity of *pvs28* (π = 0.00305 ± 0.00061) was slightly higher; however, the difference was not significant compared to that of *pvs25* (π = 0.00146 ± 0.00007) (*P* > 0.05). The number of dN in both Pvs28 and Pvs25 was higher than the number of dS. There was a positive Tajima’s D value for *pvs25*, which suggested that this gene was under balanced selection. In contrast, the Tajima's D value of *pvs28* gene was negative, which was under purification selection. Both Tajima's D values deviate from 0, which indicated natural selection was likely to be involved. The K value of *pvs25* gene ranged from 0.467 to 1.054, with a similar π value (Table 3); however, the values were not statistically significant (*P* > 0.05). The Tajima's D value of *pvs28* gene in isolates form Yingjiang was negative, while Tengchong and Nyingchi were positive (Table 4). Only one haplotype was identified in sites of Dandong and Suining.

**Table 3:** Nucleotide diversity and tests of neutrality of the *pvs25* gene in *P. vivax* isolates from six sites in China, 2009–2020

Sites	No. of isolates	S	H	Hd ± SD	K	π ± SD	Tajima’s D (*P* value)	dN
Dandong	7	1	2	0.571 ± 0.119	0.571	0.00097 ± 0.00020	1.34164 (*P* > 0.10)	0.57143
Suining	10	1	2	0.467 ± 0.132	0.467	0.00079 ± 0.00022	0.81980 (*P* > 0.10)	0.46667
Hainan	24	2	3	0.507 ± 0.093	0.623	0.00106 ± 0.00023	0.35972 (*P* > 0.10)	0.62319
Nyingchi	24	3	4	0.699 ± 0.048	1.054	0.00179 ± 0.00015	0.78580 (*P* > 0.10)	1.05434
Tengchong	205	4	7	0.489 ± 0.039	0.724	0.00123 ± 0.00011	0.12056 (*P* > 0.10)	0.72434
Yingjiang	54	3	4	0.473 ± 0.071	0.521	0.00089 ± 0.00016	− 0.42264 (*P* > 0.10)	0.52131
Total	324	5	8	0.621 ± 0.021	0.859	0.00146 ± 0.00007	0.16474 (*P* > 0.10)	0.00186

*S* number of polymorphic sites, *H* number of haplotypes, *Hd* haplotype diversity, *K* average number of nucleotide differences, *π* nucleotide diversity, *dN* number of non-synonymous substitutions

**Table 4:** Nucleotide diversity and tests of neutrality of the *pvs28* gene in *P. vivax* isolates from three sites in China, 2009–2020

Sites	No. of isolates	S	H	Hd ± SD	K	π ± SD	Tajima’s D (*P* value)	dN	dS
Nyingchi	5	4	3	0.800 ± 0.164	2.400	0.00417 ± 0.00101	1.64070 (*P* > 0.10)	2.40000	0.00000
Tengchong	206	10	21	0.841 ± 0.008	1.671	0.00966 ± 0.00031	0.86672 (*P* > 0.10)	2.97016	0.91120
Yingjiang	45	7	5	0.470 ± 0.081	1.513	0.00263 ± 0.00051	− 0.48164 (*P* > 0.10)	1.46869	0.04444
Total	256	11	22	0.485 ± 0.087	1.679	0.00305 ± 0.00061	− 0.52637 (*P* > 0.10)	2.73584	0.72924

*S* number of polymorphic sites, *H* number of haplotypes, *Hd* haplotype diversity, *K* average number of nucleotide differences, *π* nucleotide diversity, *dN* number of non-synonymous substitutions, *dS* number of synonymous substitutions

## Discussion

The emergence of drug-resistant parasites is one major impediment to global malaria control and elimination [26]. The development of TBVs may play an important role in preventing the widespread global spread of drug-resistant parasites. Pvs25 and Pvs28 represent promising candidates for TBVs [27–29]. This study was firstly reporting the genetic diversity of the *pvs25* and *pvs28* genes of *P. vivax* isolates from different geographic sites in China.

The results showed that the polymorphism of Pvs25 appeared to be more limited than that of Pvs28, which was consistent with previous reports [13, 15, 17]. In addition, 6 and 11 amino acid substitutions were identified in the Pvs25 and Pvs28, respectively, and most of the amino acid substitutions were accumulated in the EGF-2 and EGF-3 domains. Different from previous studies in Yunnan Province of China [19], a new mutation D27N of Pvs25 was first detected in Tengchong. The haplotypes of Pvs25 were consistent with the variant isolates from Myanmar, Thailand, and India [10, 17, 18]. Compared with the Sal-I strain, a T100S mutation in the EGF-2 domain of Pvs25 and another D125N mutation in the EGF-3 domain of Pvs28 were first identified. In this study, the most significant variation in the Pvs28 was the number of GSGGE/D tandem repeats. Except 57 (21.0%) isolates identified in the tandem repeats of GSGGE/D ranged of 4–8, the left 215 (79.0%) isolates were same with the Sal-I strain. Different with other studies, four isolates containing 8 tandem repeats of GSGGE/D were first identified in Tengchong. Surprisingly, the haplotypes of Pvs28 were quite different between two close sites, Tengchong and Yingjiang of Yunnan Province. The causes might be the bias of samples sizes from two sites, as the samples size of Tengchong was almost four times of Yingjiang.

One of the major obstacles to vaccine development is the genetic polymorphisms in parasite populations. Different from antigens expressed in asexual parasites, TBVs candidate genes have limited polymorphism. [12, 30, 31]. Compared with Duffy binding protein (*dbp*: π = 0.0122 ± 0.0010) [32] and merozoite surface protein 1 (*msp1*: π = 0.1193 ± 0.0.0178) [33] in the blood stage of *P. vivax*, the nucleotide diversity of *pvs25* (π = 0.00146 ± 0.00007) and *pvs28* (π = 0.00305 ± 0.00061) were relatively lower in this study. In addition, the nucleotide diversity of the *pvs25* and *pvs28* genes was analogous to that reported in countries in Southeast Asia [18], which indicated that these two genes were relatively conserved in this region. The Tajima's D values for both *pvs25* and *pvs28* deviated from 0, suggesting that these antigens might be affected by natural selection. Therefore, it was speculated that the TBVs should play a similar blocking role in malaria control and elimination in Southeast Asia.

This study revealed the amino acid substitutions of these two vaccine candidate antigens in different geographic sites in China, and the genetic polymorphisms of *pvs25* and *pvs28* were similar to the findings from Thailand and Myanmar. These results gained insight into the genetic composition of the *P. vivax* population in Southeast Asia and provided useful information for the development of effective TBVs. Previous studies have shown that the specific amino acid substitutions in Pvs25 and Pvs28 can affect parasite fitness, resulting in reduced or altered vaccine efficacy in different geographical environments [34]. This is a major obstacle to vaccine efficacy. Thus, the analysis of the genetic diversity of these two antigens in different countries and regions had a great value for evaluating the efficacy of TBVs, as vaccines used in different regions of the world may require different formulations.

## Conclusion

The key to vaccine design is to gain a comprehensive understanding of the genetic diversity of candidate antigens in different regions. *P. vivax* isolates from China demonstrated limited genetic diversity in the *pvs25* and *pvs28* genes, which could provide useful information for TBVs design and optimization.

## Data Availability

The datasets analyzed in this study are publicly available. DNA sequences of *pvs25* and *pvs28* genes were deposited in the NCBI database under GenBank accession number OP243722 to OP244045 and OP244076 to OP244347, respectively.
